# The sound of cooperation: Musical influences on cooperative behavior

**DOI:** 10.1002/job.2128

**Published:** 2016-08-09

**Authors:** Kevin M. Kniffin, Jubo Yan, Brian Wansink, William D. Schulze

**Affiliations:** ^1^ Cornell UniversityDyson School of Applied Economics and ManagementNew YorkU.S.A.; ^2^ Division of EconomicsNanyang Technological UniversitySingapore

**Keywords:** music, cooperation, experimental economics, consumer behavior, organizational behavior

## Abstract

Music as an environmental aspect of professional workplaces has been closely studied with respect to consumer behavior while sparse attention has been given to its relevance for employee behavior. In this article, we focus on the influence of music upon cooperative behavior within decision‐making groups. Based on results from two extended 20‐round public goods experiments, we find that happy music significantly and positively influences cooperative behavior. We also find a significant positive association between mood and cooperative behavior. Consequently, while our studies provide partial support for the relevance of affect in relation to cooperation within groups, we also show an independently important function of happy music that fits with a theory of synchronous and rhythmic activity as a social lubricant. More generally, our findings indicate that music and perhaps other atmospheric variables that are designed to prime consumer behavior might have comparably important effects for employees and consequently warrant closer investigation. Copyright © 2016 The Authors Journal of Organizational Behavior Published by John Wiley & Sons Ltd.

Musical resources are drawn upon for many uses. In retail establishments, for example, store managers and advertisers are careful to play songs with the goal of encouraging behaviors and attitudes that are more likely to result in greater sales (e.g., Munichor & Rafaeli, 2007; North & Hargreaves, 1998, 2003; North, Hargreaves, & McKendrick, 2000; Strick et al., 2015; Wansink & van Ittersum, 2012; Yalch, 1991). In sports, athletes tend to demonstrate greater performance while reporting less exertion when competing with their favorite music (e.g., Karageorghis & Priest, 2012). And, in romantic relationships, the phrase “mood music” reflects a tradition of suitors using music to woo the subjects of their affection (e.g., Miller, 2000).

In the same way that there are myriad reasons why people listen to music (e.g., Tarrant & North, 2000, 2001), it is also clear that people do so in a wide variety of environmental settings. Rentfrow and Gosling (2003), for example, report that people commonly listen to music when they are driving, reading, exercising, spending time with friends, and when they are alone. Indeed, the pervasiveness with which music is played appears to contribute to it being “taken‐for‐granted” (Suddaby et al., 2010) as part of the ambient environment. In our case, we are interested in the potential for music to significantly influence cooperative behavior. Our interests are complementary to recent findings that show the importance of an organization's physical features for facilitating cooperative behavior (e.g., Ashkanasy et al., 2014; Kniffin et al., 2015).

Among academic researchers, music has been closely studied for its marketing value in relation to its potential influence upon consumer behavior (e.g., Bailey & Areni, 2006; Hui, Dube, & Chebat, 1997; Morin, Dube, & Chebat, 2007); however, there has been sparse attention paid to the relevance of music for employee behavior and managerial decision making. For example, a search of the subject indices for 10 popular Organizational Behavior (OB) textbooks yields no entries for “music,” “musical,” or “song” (Champoux, 2011; Colquitt et al., 2009; Gander, Haberberg, & Rieple, 2007; George & Jones, 2008; Griffin & Moorhead, 2007; Hitt et al., 2011; Johns & Saks, 2001; Kinicki & Kreitner, 2008; Luthans, 2008; McShane & Von Glinow, 2008; Robbins & Judge, 2011). Similarly, a keyword search within a sample of high‐profile OB journals for “music” and “musical” generates no results for the *Academy of Management Journal* while articles in the *Journal of Organizational Behavior, Academy of Management Review*, and *Administrative Science Quarterly* focus on music as an industry or activity (Albert & Bell, 2002; Bougon et al., 1977; Dobrow, 2013; Faulkner & Runde, 2009; Greve, 1996; Hackman, 2003; Hirsch, 1975; Lingo & O'Mahony, 2010; Miner et al., 2001; Moorman & Miner, 1998; Peterson & Berger, 1971) instead of considering music as a potential influence on contemporary workplaces. In a partial exception from that pattern, Fritz et al. (2010) neither focus on music nor present empirical findings that involve music but they do nevertheless acknowledge that employees often listen to music on the weekends as one among many tools for recovering from the stresses of a regular workweek.

In this article, we review previous research concerning musical influences upon behavior, and we report the results of two lab experiments that test for the specific influence of music upon cooperative behavior. As with other stimuli that are designed to subtly influence behavior, our experiments are partly motivated by the fact that music is a workplace feature that can be modified with relative ease and low cost. Indeed, while many retail establishments carefully select musical programs that are designed to influence consumers' behavior during shopping visits (Wansink & van Ittersum, 2012), it is notable that the employees in those same workplaces are concurrently exposed to the company's musical selections during their shifts. We focus on studying cooperation given ample evidence that workplace teams tend to benefit when co‐workers cooperate with each other (e.g., Grant, 2013; Wilson & Kniffin, 2003). With specific respect to music, our interests are responsive to recent calls (e.g., Ashkanasy, Ayoko, & Jehn, 2014; Elsbach & Pratt, 2007) to examine ways in which office atmospherics might influence employee behavior.

Our studies draw upon two main theoretical frameworks that relate to understanding the relevance of environmental features on individual decision making within groups. Studies 1 and 2 each engage Dynamic Attending Theory (DAT) (Escoffier et al., 2010; Jones & Boltz, 1989; Large & Jones, 1999) to examine whether the synchronous activity of listening to rhythmic music will contribute significantly to cooperation while Study 2 applies Affective Events Theory (AET) (Weiss & Cropanzano, 1996) to test the question of whether mood—induced by happy music—is significantly and positive associated with prosocial decisions. In their original introduction of AET, Weiss and Cropanzano (1996, p. 40) catalogue a long list of environmental factors such as temperature, humidity, pollutants, and crowding that researchers have shown as important for influencing mood; however, our focus on happy, rhythmic music offers a chance to concurrently consider AET alongside DAT. More specifically, the intensive laboratory experiments that we present are designed to approximate workplace environments in order to help us understand (i) whether happy music facilitates cooperative behavior among co‐workers and (ii) the extent to which (if any) the induction of positive mood might explain away the relevance of the music.

## Theoretical Background and Hypothesis Development

The physical design of professional workplaces has been understudied within OB (Johns, 2010). In their review of the topic, Elsbach and Pratt (2007) reported mixed results while generally calling for more attention from researchers. More recently, Ashkanasy et al. (2014) applied the framework of AET to consider the various ways in which the physical space of workplaces can interact with social relationships. In their case, Ashkanasy and co‐authors called for researchers to consider questions such as whether “working in high‐density open‐plan workspaces triggers affective reactions such as anger and frustration that then lead to negative attitudes and poor work attitudes (low affective commitment)” (2014, p. 1180) . In related work, Kniffin et al. (2015) highlighted the specific roles that workplace eateries (e.g., cafeterias, kitchens) can have upon cooperation among co‐workers and team‐level performance. Considering the significant expenses that organizations incur for establishing and maintaining physical workspaces, it is clear that research into aspects of workplace design that are often taken‐for‐granted is warranted for assessing the degree to which firms might reasonably consider such expenses to be investments that can generate returns as a function of the design.

As part of the soundscape of many workplaces, the attention to music has mainly been limited to popular news reporting (e.g., Shellenbarger, 2012; Tierney, 2012) and qualitative research that has focused mainly on description (e.g., Martin, 1996). The discrepant levels of attention paid to music in relation to consumer behavior and employee or organizational behavior are most clear when considering retail workspaces because (i) customers and employees cohabit the locations and (ii) it is reasonable to expect that music that is selected to influence the behaviors of customers would have similar influences on employees. Beyond the specific context of retail environments, however, there are additional reasons for understanding music in relation to employee behavior. First, given that most working adults spend more time working than consuming, it is clear that studies of music should extend beyond the field of Consumer Behavior. Second, the growing literature that examines music's importance for “working out” or competing in athletics (Karageorghis & Priest, 2012) is another clear illustration that there is value gained by understanding music's role for Organizational Behavior (e.g., as part of sports teams). Third, while people often shop and consume within groups (e.g., with family members and/or friends), our interest in the relevance of music for cooperative behavior fits with the fact that workplaces tend to involve groups of people working together more intensively and regularly than people typically do as consumers.

Of course, any study of music in relation to behavior needs to distinguish between the many different characteristics that can be parsed in songs. Among other aspects, one might imagine that the words of songs can affect the most influence on people within earshot; however, more subtle mechanisms include variables such as beats per minute (Karageorghis & Priest, 2012), perceived time passage (Wansink, 1992), and rhythm (Lang et al., 2015). While previous research has tended to focus exclusively on individual‐level behavior, Lang et al.'s findings are interesting in relation to cooperation because they find that rhythmic music appears to activate “interpersonal motor coupling” through which teammates or partners are more likely to synchronize their behaviors and attitudes. While the present research builds upon Lang et al.'s findings, we will first review previous research on the relevance of music for individuals and groups in both naturalistic and lab settings before presenting novel hypotheses.

### Musical influences on individual behavior

With a partial focus on clinical outcomes, significant psychological research has been conducted to explore the role of music in relation to individual‐level thoughts, feelings, and memory (e.g., Houston & Haddock, 2007; Krumhansl, 2002; Weiss et al., 2012). For example, the clinical enterprise of “music therapy” is designed to utilize various aspects of music to help people manage a wide range of psychological challenges (e.g., Gold et al., 2009). The use of music to help people cope with stress is another common instrumental value (e.g., Ratcliffe et al., 2013), particularly in the context of music that is played for patients in a hospital setting (Devlin & Arneill, 2003) or for customers who are waiting on hold (Niven, 2015). Similarly, among marketers, the familiar use of “jingles” to help people remember store names, slogans, or phone numbers is a good example where music is intentionally used to affect behavior (Yalch, 1991). In a more concrete form of engagement, North and Hargreaves (1999) found that music kept a sample of undergraduates involved in an experimental task longer than participants who were exposed to a no‐music condition.

### Music as a group‐bonding agent

Consistent with the idea that music can serve as a functional bond within groups, recent qualitative studies have focused on the role of music among blue‐collar factory workers through the lens of relatively critical perspectives. For example, El‐Sawad and Korczynski (2007) analyzed the contents of an employee “songbook” that International Business Machines created and promoted at points between the 1920s through 1970s as part of an effort to engage employees in the company. More recently, Korczynski (2011) analyzed the use of music in a single factory he observed and concluded partly that employees with routinized work duties used music “as a dialectical cultural practice that simultaneously enacts the social order as it expresses a sense of resistance to this repetitively patterned, alienating social order” (p. 105). A foundational concern suggested, at least, by this thread of qualitative analyses (e.g., Pritchard et al., 2007) is that music in the workplace is often used as a managerial tool to extract additional productivity from workers without either (i) additional compensation and/or (ii) equivalent adjustments to the employees' working conditions or workloads.

Our specific interest in happy music as a subtle means of increasing cooperative behavior would seem to offer a benefit (i.e., more cooperation) that is broadly beneficial. For example, notwithstanding critical perspectives on the functions and effects of music in the workplace, our interest in cooperative behavior is sufficiently inclusive that worker‐focused organizations (e.g., unions) could apply the findings for their own events and environments. This interest fits with Korczynski's (2011, p. 91) acknowledgment that music can plausibly contribute to “autonomous worker communities” that “help promote collective resistance.”

Much more broadly, while the present research focuses on listening and responding to songs, the joint production of music—through choirs (e.g., Stephens, 2014), bands (e.g., Kirschner & Tomasello, 2010) or children's lesson‐groups (Schellenberg et al., 2015), for example—warrants mention as an important way through which organizations often use music to successfully enhance bonds among members. Our interests focus on the relevance of music listening because it can be done in a workplace setting relatively passively and without disruption to regular activities such as talking. While prior research shows that listening to music tends to be positively associated with happiness outside of workplace settings when people are self‐selecting the songs (Wang & Wong, 2014), it is valuable to recognize—for the sake of context—that listening to music appears to have universal importance across the lifespan. At the earliest potential points, Ivanov, Ma, and Bartsch (2009) report evidence that fetal exposure to the maternal heartbeat is an initial mechanism through which people develop affection for hearing music. Likewise, in their extensive and recent review of the varied functions associated with listening to music, Groarke and Hogan (2015) highlight the importance of music as a means for people at all stages of life, including adolescents and the elderly, to “connect” with each other.

### Lab‐based experimental research on music and cooperation

Complementary to prior qualitative research that examines music's relevance for the workplace (e.g., Korczynski, 2011), several previous lab‐based experiments have explored important aspects of music's potential relevance for workgroups. For example, Brooks and Schweitzer (2011) use the theme song from the movie *Psycho* as a priming stimulus for studying the influence of mood on negotiations, finding that anxiety‐induced participants tend to have inferior outcomes. Au, Chan, Wang, and Vertinsky (2003) present study participants with lengthy, non‐repeated tracks of “pleasant” and “unpleasant” music as a background for studying trading behavior, finding that people exposed to unpleasant music tended to take fewer risks, earn fewer rewards, and have relatively less confidence in a series of trading‐simulation studies. Wiltermuth and Heath (2009) focus on cooperation and find that (i) listening to one's national anthem can increase cooperative behavior just as (ii) singing together in synchrony with strangers can further increase cooperation within groups as measured by five rounds of experimental decision making. Similarly, Greitemeyer (2009a, 2009b, 2011) has demonstrated positive influences that music can have upon mood and behavior with a range of outcome variables including decisions made within “one‐shot” “dictator” experiments just as Krahe and Bieneck (2012) find that pleasant music can decrease aggressiveness. In effect, the experimental research on music has tended to demonstrate that music can elicit “warm‐glow giving” (Andreoni, 1990) in which people engage in altruistic acts as a function of the personal enjoyment that they gain from such actions; however, it is notable and important that all of the previous experimental research has occurred during limited periods of study (e.g., one‐shot games).

### Boundary conditions for music's group‐level contributions

Notwithstanding the beneficial outcomes and goals intended for music and identified in the preceding subsections, there are obviously boundary conditions that delimit music's positive impacts. Musical type is clearly important. For example, Lang et al. (2015) find beneficial effects of Rhythmic music when compared with Arhythmic alternatives. Across individuals within groups, variable preferences for different musical types can lead to negative outcomes if people dislike the selection of music that is played in public airspaces such as workplace settings. For example, Hawksworth, Asbury, and Millar (1997) have noted that members of surgical work groups (e.g., anesthesiologists) often indicate concern that music might impair cooperation among team members partly because a single person (e.g., the surgeon) can select stimuli that others might dislike. Particularly given the potential for certain types of music to elicit aggression in others (e.g., Mast & McAndrew, 2011; Roy, 2000), this case of concern inside the operating room illustrates some of the potential for unproductive or counterproductive disruption within work groups that can emerge when music is part of the workplace. More broadly, the common practice for retail workplaces to have their music selected by corporate‐level officials is liable to face problems as a function of both (i) the diversity of employees that one would expect to work in any large network of retail chains and (ii) the fact that customer interests rather than employee preferences will typically be the priority when retailers are planning and developing atmospheric features.

### Hypothesis development

Taking stock of prior research on the functions of music, our experiments offer substantially expanded tests of the expectations that (i) music will affect cooperative behavior within groups (Wiltermuth & Heath, 2009) and (ii) there will be a positive relationship between mood and cooperative behavior within groups (Greitemeyer, 2009a, 2009b, 2011). Given the organizational benefits that tend to accrue as a result of cooperation within groups (Grant, 2013), building upon these aspects of previous studies has clear value (Simmons et al., 2011). With respect to mood, it is notable that music is often the tool that researchers have used in order to induce mood changes (e.g., Brooks & Schweitzer, 2011). In our case, mood is not our primary interest; however, it is important to consider alongside the question of music in relation to cooperative behavior and we do so in Study 2.

Our primary question focuses on the degree to which happy music might elicit a consistent pattern of cooperative, prosocial behavior. While there are numerous ways in which music can be classified such as popular genres (e.g., Classical, HipHop, Country) or analytic variables such as beats per minute (Karageorghis & Priest, 2012), we opted to examine the importance of happy music because it is a genre‐neutral trait of music that people find broadly attractive. Comparable to Lang et al.'s (2015) analysis that individuals jointly listening to rhythmic music will be predisposed to synchronize their behavior, our interest in happy music is based on an expectation that people who hear such songs—when compared with either no music or unhappy music—will tend to enjoy the benefits of “social bonding.” In this respect, while prior research has shown that people who have an opportunity to engage in small talk with each other will be significantly more likely to cooperate with each other (Messer et al., 2007), our expectation is that happy music functions as a kind of nonverbal analogue for encouraging prosocial decision‐making.

In terms of the mechanism(s) that account for why we would expect happy music to facilitate cooperative behavior within groups, Study 1 focuses on the direct and independent relationship between listening to happy music and cooperating with others. More specifically, given the critical role of rhythm for happy music (Khalfa et al., 2008), our research draws upon DAT (e.g., Escoffier et al., 2010; Jones & Boltz, 1989; Large & Jones, 1999), which predicts that happy (and rhythmic) music will be positively associated with synchronous activity among listeners. Similar to the concept of “behavioral entrainment” that Collins et al. (2014) identify as a general explanation for individuals within groups unconsciously synchronizing their behavior with each other, DAT is rooted in the fact that humans universally tend to move in synchrony with musical beats (e.g., Patel et al., 2009). DAT is notably distinct from the more conscious “heedful” attending theory reviewed by Stephens and Lyddy (2016) and, instead, fits with a long or deep view whereby music functions as a kind of primitive, nonverbal, and nonphysical analogue to spoken language with respect to group coordination (Dunbar, 1998; Kniffin & Wilson, 2005, 2010).

Early research relating to DAT (e.g., Large & Jones, 1999) typically focused on basic patterns such as the relationship between rhythm and time perception; however, recent studies have explored different ways in which rhythm‐induced synchronized activities relate to one‐shot cooperation tasks, feelings of trust and liking, and overall perceptual and motor skills. Kokal et al. (2011), for example, found that people who successfully maintained rhythmic 10‐note drum beats with a partner tended to engage in significantly more pro‐social behavior with the partner (i.e., a confederate in the study) than people who did not synchronously drum. In contrast with Kokal et al.'s (2011) allowance for natural variation to emerge with respect to whether participants would maintain a rhythm (or not) on their own, Launay et al. (2013) instructed participants to either synchronize or “don't synchronize” the tapping of their hands in response to a tone and found that synchronized hand‐tappers demonstrated greater trust in their partners when compared with the non‐synchronized tappers. Employing similar methods, Hove and Risen (2009) found that people who were asked to tap their right index finger in synchrony with an experimenter‐partner tended to like the experimenter significantly more than either (i) people who tapped asynchronously with the experimenter and (ii) people who were asked to tap in synchrony with a metronome with the experimenter nearby in the room.

In a more physical study focused on synchrony, Valdesolo et al. (2010) asked people to run in pairs with or without synchronizing their strides and found that synchronized runners tended to demonstrate greater perceptual and motor skills in a post‐run experimental task. Interestingly, Valdesolo et al. (2010) speculated that simply watching synchronized runners might elicit similar outcomes when compared with how people would respond after watching non‐synchronized runners but they did not test that question. Our focus on music in the ambient environment effectively offers a test of Valdesolo et al.'s speculation because we are interested to see whether relatively passive engagement with happy, rhythmic music facilitates positive outcomes that are comparable to those found through active tasks such as drumming, tapping, or running in synchrony. An important practical benefit of our focus is that listening to music is a much more naturalistic activity for people in the workplace than the more active foci for prior studies.

Our interests to examine the influence of happy music in a relatively extensive series of economic decisions complements prior studies that have examined other outcome variables. We focus on cooperation, though, as a kind of synchronous activity because it logically extends previous research and is clearly important in contemporary organizations (e.g., Grant, 2013; Kniffin, 2009). Against that backdrop, the public goods experiment that we conducted offers a clear test of cooperation within groups. Hypothesis 1, therefore, predicts that happy music will effect synchronized activity among group members that will result in cooperative behavior in the context of a public goods dilemma.

Hypothesis 1 Happy music will contribute to significantly higher levels of cooperative behavior.

## Study 1

### Participants and procedures

We recruited participants on the campus of a private university in the Northeastern United States to join one of four experimental sessions with students recruited from an undergraduate business course.

With a between‐subjects design, 78 participants (27 women) were randomly assigned into one of two conditions in which they were exposed to either Happy Music (33 participants) or Unhappy Music (45 participants).

In order to assess the potential influence of demographic variables, we asked participants to indicate their gender and register their age within one of four categories: 18–22 (1), 23–29 (2), 30–39 (3), and 40 or more years (4). We also asked participants to report their major or field of specialization in light of previous research showing that public goods experiments tend to elicit significantly more selfish responses from Economics students when compared with students pursuing other majors (e.g., Wang et al., 2011).

In each case, once participants were welcomed to participate in the study and provided informed consent, we turned on the Happy or Unhappy Music for the respective sessions. Through a centralized audio system, we were able to ensure that the volume was uniformly audible—at a comfortable level—for all of the experiment's participants.

Participants' desks in the lab included privacy hoods that helped to ensure that each person's decisions were free from visual knowledge of the decisions that were made by people sitting near them. Participants' attention during the decision‐making tasks was focused on the computers through which we provided decision prompts through software that is maintained through the University of Virginia's public‐domain VECON Lab website (veconlab.econ.virginia.edu).

In addition to partial course credit, participants earned cash compensation that was contingent upon the decisions that they and their fellow participants made during the course of the experiment. Average cash compensation for our sessions was $5 for each participant, with a range from $3 to $7. The experimental sessions lasted an average of 20 min with all of them completed between 18 and 22 min. An important benefit of our experiment's use of contingent rewards is that the existence of material stakes—in this case, above the wage rate for most employees working in retail workplaces—engages realistic decision making (Davis & Holt, 1992).

### Independent variable

For stimuli, we selected a set of four “Happy” songs (“Yellow Submarine” by the Beatles; “Walking on Sunshine” by Katrina and the Waves; “Brown Eyed Girl” by Van Morrison; and, the theme song from “Happy Days”) that total to 12 min of run‐time as well as a set of “Unhappy” songs by less familiar “heavy metal” bands (“Smokahontas” by Attack Attack! and “You Ain't No Family” by Iwrestledabearonce) that total to 8 min of play. We tested the stimuli with a sample of 44 undergraduate students who listened to two songs from one of the two conditions. Average ratings for the two groups of songs varied significantly according to each of the measures that we assessed. Specifically, for the Happy songs, raters indicated significantly more agreement with the statements that “the song was happy” (*F* = 530.7, *p* < .001) and “I liked the song that I just heard” (*F* = 49.6, *p* < .001) and significantly more disagreement with the statement that “the song was angry” (*F* = 303.0, *p* < .001). Raters also indicated significantly greater “warmth” (Fiske et al., 2002) when asked “How ‘cold’ or ‘warm’ do you consider the song that you just heard” (*F* = 136.0, *p* < .001).

Three measures that we assessed in relation to DAT also showed significant differences. Specifically, and consistent with Khalfa et al.'s (2008) recognition that rhythm is a critical aspect of Happy music, raters for the Happy songs agreed significant more with the statements: “The song was very rhythmic” (*F* = 60.6, *p* < .001), “The song made me ‘move to the beat’ (e.g., tap my foot, leg, hand, or nod my head)” (*F* = 29.2, *p* < .001), and “The song would be good dance music” (*F* = 40.5, *p* < .001).

### Dependent variables

#### Cooperative performance

To measure cooperation as a function of our musical conditions, we administered a traditional public goods experiment known as the Voluntary Contribution Mechanism (VCM) (e.g., Isaac & Walker, 1988, Rondeau et al., 2005, Wiltermuth & Heath, 2009). In each round of this experiment, participants receive a fixed sum of money across an unspecified number of iterations or “rounds” during which they are asked what portion to allocate to a group pool and what portion to retain for private use. In our case, participants were allocated a total of 10 symbolic tokens—corresponding to actual cash values—in each round for them to allocate as they wished with 0 to 10 tokens being allocated to either the group or private accounts.

As part of the VCM that is designed to incentivize and reward cooperation, we set the parameters so that contributions to the group pool were multiplied by 1.5 before being split evenly by the software program among the three people within each decision‐making group. Participants in each of our sessions made decisions interactively with a fixed but randomly assigned set of two other people for the duration of the rounds. Participants were not informed of the identity of their partners at any point during the experiment (e.g., they could have been interacting with their immediate neighbor or someone at the opposite corner of the room). Participants also did not talk with each other during the experiment partly because previous tests of the VCM have found that even relatively small amounts of casual conversation immediately prior to the VCM tends to increase people's contributions to the public good (e.g., Messer et al., 2007). While these conditions do not replicate naturalistic environments, they do provide controlled and validated measures of cooperative behavior (Henrich et al., 2005).

Each session involved 20 rounds of the same public goods dilemma, but participants were not informed of the specific number of rounds until we were done in order to avoid “last round effects” (e.g., Gintis, 2000) where the incentive for retaining all of one's money would significantly increase because—in this kind of *ad hoc* group (Hollenbeck et al., 2012)—there would be no prospect of potential reciprocity if someone were to “cheat” the group and engage in purely selfish behavior (Axelrod, 1984). For purposes of our analysis, we focus on the individual‐level contributions made by each participant across all 20 rounds. While it is noteworthy that only the 78 observations from Round 1 can be considered fully independent because individual decisions in rounds 2 through 20 will be influenced by the outcomes of previous rounds, our analysis is able to consider 1560 decisions (78 participants × 20 rounds) to cooperate or not in relation to a public good that was tied to the actual compensation that participants were allocated. In this respect, we follow common analytical approaches for the VCM (Messer et al., 2013, Montmarquette et al., 2004, Wiltermuth & Heath, 2009).

To specify the range of options that exist in any given round, each group is able to pool between 0 and 30 tokens in light of the range whereby each of the three participants can commit between 0 and 10 tokens individually. In a scenario where each member contributes 10 tokens, the design of the experiment—like many naturalistic dilemmas that reward cooperation among partners (e.g., Axelrod, 1984)—generates a payout of 15 tokens for each participant because contributions to the pooled or group account are multiplied by 1.5 before being divided up for the three participants. In contrast, when participants uniformly make no contributions to the public good, each person is limited to the 10 tokens that they choose to retain rather than cooperatively invest in the group.

### Results

Table 1 shows no significant differences between the two treatment groups with respect to the key demographic variables of age, gender, and whether the person was majoring in Economics. The lack of demographic differences between treatments fits with our goal of random assignment across experimental conditions. With respect to the dependent variable, snapshot analyses of individuals' contributions to the public good as measured through rounds 1, 5, 10, 15, and 20 show a consistent nominal pattern whereby people listening to Happy music contributed more.

**Table 1:** Descriptive statistics and comparisons by treatment for Study 1

	Unhappy music		Happy music		*T*
Variable (Scale)	*M* N = 45	*SD*	*M* N = 33	*SD*	*Happy and unhappy*
Age (1–4)	1.044	0.298	1.061	0.242	0.256
Male (0/1)	0.622	0.490	0.697	0.467	−0.679
Econ Major (0/1)	0.889	0.318	0.939	0.242	0.764
VCM 1 (0–10)	5.044	3.418	5.485	3.438	0.561
VCM 5 (0–10)	3.822	3.816	6.091	3.835	2.589^**^
VCM 10 (0–10)	3.178	3.910	3.697	3.245	0.622
VCM 15 (0–10)	2.511	3.727	2.818	3.566	0.366
VCM 20 (0–10)	2.333	3.879	2.485	3.251	0.182

* *p* < 0.05;

** *p* < 0.01;

*** *p* < 0.001.

Notably, only one of the five snapshots reported in Table 1 is statistically significant according to *t* tests; however, the sustained pattern of nominally predicted results encouraged us to conduct a more extensive study with more methodological power. To supplement Table 1, though, we can note here that Cohen's *d* for the respective snapshots are .13 (Round 1), .59 (Round 5), .14 (Round 10), .08 (Round 15), and .04 (Round 20). As measures of effect size that help make sense of statistical non‐significance (e.g., Dingemans & Henkens, 2014) that take into account Mean differences, Standard Deviations, and cell‐sizes, the uniformly positive values for Cohen's *d* reinforce our recognition that the simple difference in Means displayed in Table 1 warranted the more extensive and powerful approach that we undertook for Study 2.

## Study 2

With the benefit of results gained through Study 1, we expanded the research design for Study 2 to include a third no‐music Control condition as well as a set of repeated within‐subject mood measures. The no‐music Control was included to allow us to interpret the differences we found in Study 1. For example, if we found that participants in the Control condition cooperated as much as the people in the Happy condition, then we could infer that there was no benefit to the Happy music but, instead, simply a negative effect of the Unhappy music. On the other hand, if participants in the Control cooperated less than the Happy‐music participants, we could infer that Happy music functioned as a positive intervention that increased cooperative behavior.

With respect to mood, we were motivated by prior research showing relationships between music and mood (e.g., Brooks and Schweitzer, 2011) to consider whether mood might be positively correlated with the dependent variable in our experiment. In effect, while H1 presumes a “warm‐glow” effect (Andreoni, 1990) for Happy music, Study 2 explores the potential role of mood when accounting for any overarching relationship between our experimental conditions and contributions to the public good. More specifically, our expectation is that better or higher mood levels will positively correlate with the dependent variable of contributions to the public good. There does exist previous research that has examined this relationship between mood and prosocial behavior but the evidence has been mixed (e.g., Hertel et al., 2000; Isen & Baron, 1991). Our consideration of mood in relation to the dependent variable is necessarily limited to examining non‐causal relationships because—unlike the exogenous independent variable of musical condition—mood is endogenous to Study 2's research design.

More conceptually, Study 2 permits us to consider AET (Weiss & Cropanzano, 1996). Through the AET framework, one would expect that it is the induction of improved moods that significantly influences the relationship that we found in Study 1 between Happy music and cooperative behavior. Thompson et al. (2001) illustrate this approach with their finding that the apparent positive relationship between listening to classical music and performing better in spatial tests—known as the “Mozart effect”—is best and sufficiently explained on closer analysis by the significantly higher moods and arousal levels generated by the music. Consistent with Thompson et al.'s analysis, it is notable that much of the research concerning the relevance of mood on work‐relevant decisions and behavior tends to treat the stimulus for mood as relatively unimportant as a matter of regular practice. For example, in their extensive literature review on the concept of “group affect,” Barsade and Knight (2015) make no reference to music and, more notably, they consistently use the passive voice (e.g., “positive mood had been induced”) in a way that ignores the environmental factors that successfully modified the participants' emotions.

Our concurrent consideration of the relationships between Happy music and mood with the outcome variable of cooperative behavior fits with Herrbach's (2006, p. 633) description of AET as a framework for expecting both affect and environmental workplace features to be important. Similarly, Fisher and Ashkanasy (2000, p. 124) credit AET as an approach that considers both the antecedents and consequences of affect for understanding behavior. While Hypothesis 1 proposes that Happy music contributes significantly to cooperative behavior as an antecedent factor, Hypothesis 2 focuses on the expected positive relationship between higher mood and prosocial behavior.

Hypothesis 2 Higher mood will be associated positively with cooperative behavior.

### Participants and procedures

Except for those details specified above regarding a Control condition without music and the integration of a mood measure, Study 2 follows the same procedures as described for Study 1.

Employing a between‐subjects design, 188 participants (75 women) were recruited from the same population as Study 1 (with no overlap in participation) and randomly assigned into one of three conditions in which they were exposed to either Happy Music (n = 60) or Unhappy Music (n = 69) or the no‐music Control (n = 59). Ninety‐three per cent of participants were between 18 and 22 and 6 per cent between 23 and 29.

#### Mood

We used the four‐item mood short form (Peterson & Sauber, 1983) in order to assess mood before, during, and after the experiment. Asked to rate their agreement on a scale of 1 (strongly disagree) to 5 (strongly agree), participants were presented with the statements: “Currently, I am in a good mood,” “As I answer these questions, I feel cheerful,” “For some reason, I am not very comfortable right now,” and “At this moment, I feel edgy or irritable.” Values for the third and fourth questions are reverse‐coded, and the sum of the four measures comprises a score for mood in which higher numbers reflect more positive moods.

In order to measure mood changes across the course of our experimental sessions, we assessed mood immediately after gaining informed consent (Mood 1), immediately prior to the first round of the VCM (Mood 2), in between Rounds 10 and 11 of the VCM (Mood 3), and immediately after the final round of the VCM (Mood 4). Reliability analyses indicated that each of the Mood measures were internally coherent (Mood 1: α = .75; Mood 2: α = .78; Mood 3: α = .76; Mood 4: α = .77).

## Results

As illustrated in Table 2, we found significantly and persistently higher levels of cooperative behavior by participants who were played Happy music when compared with the other two conditions. Additionally, the post‐hoc Tukey analyses reported in the right‐most columns of Table 2 confirm the success of our random assignment of individuals across the three experimental conditions because there are no significant demographic differences. Table 2 selectively presents snapshots from Rounds 1, 5, 10, 15, and 20 of the VCM and shows that Happy music generates significantly more cooperative behavior when compared with the control in each of the selected rounds with the sole exception of Round 15. For our four measures of mood, Table 2 shows that initial mood measures—assessed immediately after gaining informed consent—were randomly distributed among the treatments while the effect of Unhappy music persisted strongly upon mood throughout the rest of the experiment when compared with the Control.

**Table 2:** Descriptive statistics and comparisons by treatment for Study 2

	Unhappy music		Happy music		Control		*T*	*T*
Variable (Scale)	*M* N = 69	*SD*	*M* N = 60	*SD*	*M* N = 59	*SD*	*Control and unhappy*	*Control and happy*
Age (1–4)	1.145	0.459	1.067	0.250	1.034	0.181	1.89	0.54
Male (0/1)	0.681	0.466	0.617	0.486	0.492	0.500	2.20	1.40
Econ Major (0/1)	0.145	0.352	0.150	0.357	0.051	0.220	1.69	1.69
VCM 1 (0–10)	4.710	3.241	5.766	3.202	4.678	3.781	0.06	1.85
VCM 5 (0–10)	3.971	3.208	6.483	3.170	4.424	3.500	−0.78	3.41***
VCM 10 (0–10)	3.319	3.441	5.467	3.615	3.695	3.720	−0.59	2.70*
VCM 15 (0–10)	4.275	3.617	4.533	3.432	3.237	3.385	−1.68	2.03
VCM 20 (0–10)	2.942	3.543	4.983	4.015	2.271	2.999	1.07	4.17***
Mood 1 (4–20)	14.507	3.225	14.367	3.178	14.814	2.892	0.56	0.80
Mood 2 (4–20)	13.232	3.477	14.750	3.160	14.763	2.849	2.70**	0.02
Mood 3 (4–20)	12.232	3.663	14.850	3.129	14.356	3.010	3.55***	−0.88
Mood 4 (4–20)	12.284	11.269	14.333	3.448	14.322	2.837	3.170**	−0.020

* *p* < 0.05;

** *p* < 0.01;

*** *p* < 0.001.

**Figure 1:**
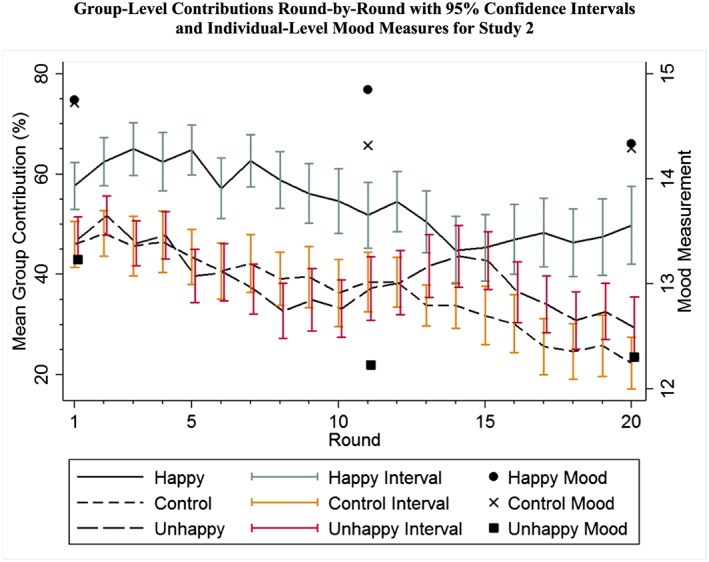
Group‐level contributions round‐by‐round with 95 per cent confidence intervals and individual‐level mood measures for Study 2 [Colour figure can be viewed at wileyonlinelibrary.com].

As depicted in Figure 1's round‐by‐round line graph of group‐level contributions, the individual‐level patterns reported in Table 2 are clear whereby people listening to Happy music tend to generate higher contributions to the public good. More specifically, Figure 1 shows the variable degrees of cooperation—as a function of the experimental condition—according to the average sum of individual contributions to each group across rounds. These findings, which show that Happy music significantly and positively influences cooperative behavior, support Hypothesis 1.

Table 3 offers additional perspective on the patterns in Figure 1 through three models that each account for the effect of Round on individual contributions to the public good. Round is important to consider because it is normal for participants to gradually decrease their cooperation—as Figure 1 shows—in multi‐round versions of the VCM (e.g., Isaac & Walker, 1988). As complements to the snapshot analyses presented in Table 2 for five of the rounds (1, 5, 10, 15, and 20), the first model in Table 3 shows that there exists a strong and significant treatment effect of Happy music even when Round is considered. The second and third models isolate, respectively, Rounds 1–10 and 11–20 and show that the treatment effect for Happy music continues across both halves of the experiment. In all three of the models, Round is significant and negative because there is a gradual decrease in contributions across the full sample. The main point of Table 3, however, is to show that even with the gradual decay in contributions associated with Round, there is nonetheless a consistent significant treatment effect for Happy music.

**Table 3:** Results of regression analyses for predicting individual‐level contributions for Study 2

	(1) Contributions 1–20	(2) Contributions 1–10	(3) Contributions 11–20
Happy music	1.714*** (0.435)	1.671*** (0.469)	1.757*** (0.473)
Unhappy music	0.165 (0.435)	−0.239 (0.458)	0.569 (0.438)
Round	−0.101*** (0.000)	−0.135*** (0.285)	−0.118*** (0.031)
Constant	4.784*** (0.000)	5.092*** (0.364)	4.39*** (0.563)
*N*	3760	1880	1880

*Note:*

*SE* in parentheses. Group‐clustered standard errors were used to calculate *p* values.

* *p* < 0.05;

** *p* < 0.01;

*** *p* < 0.001.

Focusing on the role of mood in our findings, Figure 1 as well as Table 2 show that there is a clear and consistent treatment effect whereby Unhappy music appears to elicit worse moods than both the Happy music and control conditions. Additionally, while Table 2 and Figure 1 show a treatment effect of Happy music upon cooperative behavior, Table 4 presents two models that examine the relevance of mood independently of the three music‐related conditions. Model 1 considers each of the individuals' contributions in each round as the dependent variable and takes an average of our second and third mood measures for predicting Rounds 1 through 10 and an average of our third and fourth mood measures for predicting Rounds 11 through 20. Model 2 considers simply the average contribution across all 20 rounds as the dependent variable and takes an average of the second, third, and fourth mood measures as the independent variable. Our analyses of the role of music are independent from the regressions that we presented in Table 4 because the musical treatment is exogenous to our experimental design whereas mood—presumably influenced by the musical treatments—is endogenous; consequently, the two variables should not be considered to be equivalent independent variables with respect to the outcome measures of cooperative behavior. It is notable that Model 1 yields a statistically significant influence of mood but the effect size is approximately 1/17 of the effect that Happy Music is demonstrated to generate (as reported in Table 3), and when the number of observations is reduced as entailed by the analytical approach in Model 2, the importance of Mood is non‐significant. These findings offer support in relation to Hypothesis 2 while concurrently indicating that Happy music—as per H1—retains an independently important influence upon cooperative behavior.

**Table 4:** Results of regression analyses for predicting individual‐level contributions using mood for Study 2

	(1) Contributions 1–20	(2) Average individual contribution
Mood	0.100*** (0.053)	0.096 (0.059)
Round	−0.099*** (0.014)	—
Constant	3.996*** (0.761)	3.016*** (0.841)
*N*	3740	186

*Note:*

*SE* in parentheses. Group‐clustered standard errors were used to calculate *p* values. Model 1 is based on data from 187 participants because mood records were missing for one participant from the full sample. Model 2 is based on data from 186 participants because there was incomplete mood information for one participant.

* *p* < 0.05;

** *p* < 0.01;

*** *p* < 0.001.

**Figure 2:**
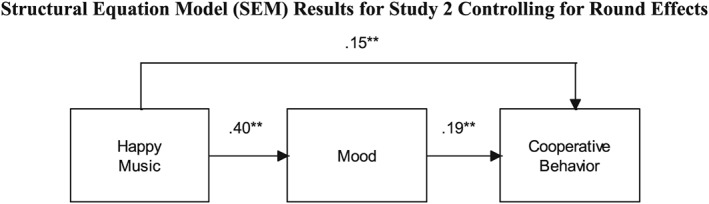
Structural equation model (SEM) results for Study 2 controlling for round effects

As a complement to our direct tests of Hypotheses 1 and 2, a structural equation model (SEM) with standardized coefficients (Mathieu & Taylor, 2006) was also used to examine the degree to which mood mediates the relationship between Happy music and cooperative behavior while controlling for Round. Figure 2 illustrates that Happy music was positively related to Mood (*β* = .40, *p* < .01) and, further, Mood was positively related with Cooperative Behavior (*β* = .19, *p* < .01). The total indirect effect of the path between Happy music, Mood, and cooperative behavior is also significant (*β* = .08, *p* < .01) and compares with the total direct effect of the path between Happy music and cooperative behavior (*β* = .15, *p* < .01). In other words, Mood does not explain away the relevance of Happy music in relation to the cooperative behavior because there are significant direct and indirect effects.

## Discussion

Given the pervasiveness of music and its careful use by marketers to “pipe” music into places (e.g., supermarkets, elevators, lobbies, exercise gyms, and hair salons) where people might “mindlessly” be influenced (e.g., Wansink, 2006), greater attention should be paid to the potential influence of music upon workplace behavior. While music in the workplace is stereotypically associated with the kind of “routinized,” blue‐collar work where the role of music has been qualitatively studied—with a critical perspective (e.g., Korczynski, 2011), the example of surgeons that we discussed helps to illustrate the broader potential value of studying and using music in the workplace. No conflict between marketing and managerial interests exists in the operating room because patients are typically under heavy sedation; however, once that unique condition is removed, it is clear that music in retail workplaces has the potential to generate different effects among workers than customers. Among non‐retail environments, the example of the operating room also illustrates the potential for disagreements and conflicts to emerge among co‐workers with respect to the type of music that is played in a workplace. These are among the aspects of music that highlight how organizationally focused research can add to findings that have, to date, been shown by research focused on the influence of music for marketing interests.

Through the pair of studies that we conducted, our findings draw upon both DAT (e.g., Escoffier et al., 2010; Jones & Boltz, 1989; Large & Jones, 1999) as well as AET (e.g., Weiss & Cropanzano, 1996). With respect to DAT, we show that happy music—including its key feature of rhythm (Khalfa et al., 2008)—is consistently and positively associated across a 20‐round lab study with cooperative behavior within groups. Concerning AET, we also find that mood is positively associated with cooperative behavior but does not explain away the independent function of happy music. This latter finding of a mediated role of mood strikes the middle ground between the view that stimuli such as happy music simply induce moods that are favorable to cooperative behavior (e.g., Thompson et al., 2001) and other findings of no relationship between mood and cooperation (e.g.,. Hertel et al., 2000).

Among the limitations of the present experiments that future research can consider further, the music that we selected could be refined to provide more subtle contrasts. For example, it seems plausible that happy songs with collectivist themes—and consistent references to the first‐person plural “we”—might elicit more cooperation than happy songs with individualist themes (Sela et al., 2012). Similarly, our analysis omits potentially important variables such as individual‐level personality measures that conceivably would moderate the relationship between Happy music and cooperative behavior. On a related topic with respect to Hypothesis 2, it is plausible that more diverse measures of mood as well as discrete emotions (e.g., Gooty, Gavin, & Ashkanasy, 2009) would have offered more insight than the 4‐item Mood Short Form that we utilized.

Given that our sample's demographic traits were equivalent across experimental conditions—as intended by our random assignment, the risk that personality differences spuriously accounted for the effects that we reported seems minimal; however, future research concerning the relationships between music, behavior, and mood would benefit from recognizing that some industries and firms have employees with relatively unique personality profiles. Among additional differences from the current lab experiments, future research would also certainly benefit from conducting studies with naturalistic settings in which participants would be able to select their own music (e.g., to be played through headphones). Notably, Oldham et al. (1995) examined the influence of office workers wearing or not wearing headphones to hear music; however, their study importantly does not consider differences in the musical style or content.

With respect to the question of whether the repetitiveness of the music that we presented to participants in both the Happy and Unhappy music conditions was important, future research can address that question by comparing comparably happy and unhappy music for uniform amounts of time whereby the music loops several times in one pair of experimental conditions and does not loop in the other non‐repeating pair. Of course, future research should also be attentive to limitations that are associated with any studies involving music—including the present studies—whereby it is imaginable that individuals' familiarity with—and relative degrees of liking for—any specific type of music can disproportionately drive results. As with the potential concern about personality differences being omitted, the random assignment of individuals in our experiments offers a reasonable basis to expect non‐significant differences among participants in the three experimental groups with respect to their orientation to the two types of music we presented. While the relative unfamiliarity of the unhappy music that we played constitutes a limiting confound to the current studies, we can highlight that (i) the present studies help to establish the presence of important relationships among music, mood, and cooperative behavior while (ii) future research can further explore the parameter space of musical types including but not limited to familiarity in finer detail.

A final limitation to acknowledge is that notwithstanding the benefits that are gained by the kind of experimental approach that we undertook and discussed; and, further, notwithstanding the fact that the present studies run between 4 and 20 times the number of rounds of previous experiments involving music, there is clearly value to field‐based research that would examine the importance of music in relation to cooperative behavior. In other words, given that our 20‐round studies are still significantly shorter than a single work shift would be in a naturalistic work environment, future field research will be important that examines the differential influence of music in different types of workplaces. For example, while it is our expectation that music in customer‐facing environments will tend to be relatively happy, we also expect that music selected by workers in workplaces that are free of customers (e.g., automobile garage stalls) will be more varied and, sometimes, includes different kinds of unhappy music. It would also be valuable for field research concerning the influence of music in the workplace to expand the scope of “outcome” variables beyond cooperative behavior. For example, in light of the relatively critical perspectives that we described above (e.g., El‐Sawad & Korczynski, 2007; Korczynski, 2011; Pritchard et al., 2007), it would be useful to see if employee satisfaction—independent of any positive or negative effects for the employer—might vary significantly as a function of the type of music that is played at work. Indeed, given that prior research shows that high‐intensity noise tends to generate negative health effects alongside psychological dysfunction (e.g., Cohen et al., 1981), it is imaginable (as a corollary) that happy music offers positive individual‐level benefits for employees who are exposed to the songs.

In stark contrast with the close focus that marketing researchers as well as psychologists, more generally, have committed to understanding the myriad roles of music, primarily in relation to individual behavior during shopping trips, the dearth of studies focused on employee impacts warrants closer attention. Through the present research, we identify important patterns that include a clear and consistent effect of happy music upon cooperative behavior. Building upon our focus on music, it would be valuable for future research to examine the relevance of other environmental features highlighted by marketing researchers (e.g., favorable smells: Doucé & Janssens, 2013) for potential influence on employee behavior in retail and non‐retail workplaces.

## Conclusion

Cooperative behavior among teammates within a group tends to yield positive, synergistic outcomes when group interests are aligned with broader organizational goals (Campbell, 1994; Grant, 2013; Kniffin, 2009). Our overarching interest in the relevance of music on cooperative behavior draws upon theoretical frameworks that are focused on the potential for environmental features to influence prosocial behavior directly and independently—as predicted by the DAT—as well as indirectly via mood as expected by the AET. More practically, while the use of music in retail workspaces partly inspired our research, our lab design focuses on a proximate dimension of music (happy/unhappy) that we expect to be relevant for employees in retail and non‐retail organizational environments. Given the contingent stakes that informed participants' decisions, our experimental design highlights a concrete way in which atmospheric variables that are typically studied in relation to consumers might independently influence employee behavior. As a complement to recent calls for more attention to the physical features of workplace environments, our findings draw attention to the importance of soundscapes in relation to employee behavior.
